# Cerebrolysin ameliorates ketamine-mediated anxiety and cognitive impairments via modulation of mitochondrial function and CREB/PGC-1α pathway

**DOI:** 10.1186/s13041-025-01255-1

**Published:** 2025-11-07

**Authors:** Leila Hosseini, Nasrin Abolhasanpour, Fatemehsadat Seyedaghamiri, Parisa Hassanzadeh, Parviz Shahabi, Vida Mafikandi, Parinaz Kalejahi, Mojgan Rajabi, Zahra Shokri, Ali Fakhari

**Affiliations:** 1 Research Center of Psychiatry and Behavioral Sciences, Tabriz University of Medical Sciences, Tabriz, Iran; 2 Research Center for Evidence-Based Medicine, Iranian EBM Center: A Joanna Briggs Institute (JBI) Center of Excellence, Tabriz University of Medical Sciences, Tabriz, Iran; 3 Neuroscience Research Center, School of Medicine, Shahid Beheshti University of Medical Sciences, Tehran, Iran; 4 Department of Physiology, Faculty of Medicine, Tabriz University of Medical Sciences, Tabriz, Iran; 5 Neurosciences Research Center (NSRC), Tabriz University of Medical Sciences, Tabriz, Iran

**Keywords:** Anxiety, Cerebrolysin, Memory, Mitochondrial function, Schizophrenia

## Abstract

Schizophrenia is known as a complex and devastating mental disorder due to its profound impact on individuals, families, and society. Emerging evidence proposes that mitochondria play a central role in schizophrenia. Here, we investigated whether cerebrolysin (CBL) can alleviate anxiety-like behaviors and cognitive deficits through a mechanism involving the CREB/PGC-1α pathway. In this study, 30 male BALB/c mice were randomly assigned to three different groups: Control, Ketamine, and Ketamine + CBL. Intraperitoneal injection of ketamine was performed at 20 mg/kg for 14 consecutive days. CBL was delivered intraperitoneally at 2.5 mL/kg once daily for seven days, starting from the 8th day to the 14th day of the experiment. The novel object recognition and elevated plus-maze tests were used to assess episodic-like memory and anxiety, respectively. Hippocampal tissue was examined not only for alterations in mitochondrial activity, encompassing ATP production and levels of reactive oxygen species (ROS), but also for estimating CREB, p-CREB, and PGC-1α protein levels. Behavioral results indicated that treatment with CBL reversed anxiety-like behavior and cognitive dysfunction caused by ketamine. Additionally, ketamine increased the production of ROS and reduced ATP levels in the hippocampus, while CBL treatment restored these changes. Furthermore, CBL therapy upregulated the hippocampal expression of the proteins CREB, p-CREB, and PGC-1α compared with the ketamine-treated animals. It is speculated that treatment with CBL can attenuate ketamine-induced cognitive deficits and anxiety-like behaviors through the upregulation of the CREB/PGC-1α pathway and the improvement of mitochondrial function.

**Supplementary Information:**

The online version contains supplementary material available at 10.1186/s13041-025-01255-1.

## Introduction

Schizophrenia (SCZ), a multifactorial psychiatric disorder with high heritability, affects about 1% of the world’s population [1]. SCZ is a chronic and disabling psychiatric disorder characterized by the presence of positive and negative symptoms, along with impairments in cognitive function [2]. The current hypothesis of SCZ suggests that positive symptoms, such as hallucinations, delusions, and abnormal motor behavior, are associated with hyperactive dopaminergic activity mediated by dopamine D2 receptors in subcortical regions, including the striatum and nucleus accumbens. Conversely, negative symptoms (e.g., avolition, anhedonia, alogia, and asociality) and cognitive deficits (e.g., impairments in working memory, cognitive flexibility, and attentional processing) are linked to dopamine activity mediated by dopamine D1 receptors [3]. Moreover, anxiety and depressive symptoms are commonly observed in various phases of SCZ, which can adversely affect social interaction and increase the risk of relapse and suicide [4].

N-methyl-D-aspartate (NMDA) receptors, which are ionotropic glutamate receptors, play a central role in the pathophysiology of SCZ, with evidence pointing to their hypofunction as a key contributor to the disorder’s development and symptoms [5, 6]. Noncompetitive antagonists of the NMDA receptor, including ketamine [7], phencyclidine [8], and dizocilpine [9], are frequently used in rodent models to induce NMDA receptor hypofunction. It is well known that sub-chronic and intermittent administration of sub-anesthetic and low doses of ketamine leads to cognitive, behavioral, neurochemical, and metabolic alterations in rodents that effectively reproduce changes observed in the brain of individuals with SCZ [10, 11]. The management of SCZ, including prevention, diagnosis, and treatment, has increasingly attracted the attention of clinicians [12].

The human brain, making up roughly 2% of the body’s mass, is a highly energy-demanding organ, consuming nearly 20% of the body’s overall energy [13]. It is not surprising that metabolic dysfunction and a mismatch between energy supply and demand are involved in several neurological and psychiatric disorders [14]. Mitochondria are the primary energy source for neurons and play a crucial role in various neural processes, including neuronal growth, synaptic plasticity, and neurotransmission [15]. Mitochondria also regulate various cellular processes, including apoptosis, reactive oxygen species (ROS) generation, and calcium influx. Impaired mitochondrial function significantly contributes to the neurobiology of SCZ [16]. Reports have shown decreased ATP levels, increased ROS production, and changes in cytochrome c release, mitochondrial translocase expression, and lipid peroxidation in patients with SCZ [17, 18].

CREB (cyclic adenosine monophosphate response element-binding protein) is a vital transcription factor involved in multiple physiological processes in the central nervous system (CNS), including neurotransmission, neuronal survival, nervous system development, synaptic plasticity, learning, and memory [19]. Disruption of CREB-mediated signaling has been associated with a variety of disorders in the CNS, including SCZ [20]. CREB is also a critical transcription factor that regulates the peroxisome proliferator-activated receptor gamma coactivator 1-alpha (PGC-1α) expression [21]. PGC-1α, as the central regulator of mitochondrial function, is expressed in tissues with high energetic demands, including the heart, brown adipose tissue, and the brain (olfactory bulb, hippocampus, cerebral cortex, and striatum) [22]. PGC-1α can regulate the transcription of mitochondrial biogenesis genes through direct interactions with PPARs, estrogen-related receptors, and NRF-1 and NRF-2, key nuclear respiratory factors that govern the expression of genes essential for the metabolic functions of mitochondria [23]. In addition, PGC-1α is a potent regulator of ROS metabolism [24]. PGC-1α expression has been studied in cortical tissue from postmortem SCZ patients. The results indicate a decrease in its expression and Nrf1 expression [25].

Cerebrolysin (CBL) is a neuropeptide purified from pig brain with a combination of peptides and amino acids with a low molecular weight and capable of effectively crossing the blood-brain barrier [26]. It contains a mixture of neurotrophic factors, such as BDNF, nerve growth factor (NGF), and ciliary neurotrophic factor (CNTF) [27]. In addition, it has demonstrated neuroprotection effects in various pathological conditions, including SCZ, seizures, intracerebral hemorrhage, Parkinson’s disease, and spinal cord injury [27–31]. The neurotrophic effect of CBL interferes with the formation of free radicals, inflammatory changes, and monoamines [32]. A study has shown that CBL improved cerebral ischemic injury by activating the CREB/PGC-1α pathway and reducing inflammation [32].

Although available antipsychotic drugs effectively alleviate positive symptoms, these interventions do not effectively manage the negative and cognitive aspects, highlighting the need for the development of more comprehensive therapeutic strategies. Recent studies have implicated the function of mitochondria and metabolic disorders in the underlying mechanisms of SCZ. Hence, gaining deeper insight into mitochondrial dysfunction in SCZ could open new avenues for therapeutic interventions that can target symptoms inadequately managed by current antipsychotic medications alone. Few studies have investigated the effects of CBL in SCZ, and the precise mechanisms of action of CBL still need a comprehensive evaluation. Therefore, this study was designed to examine its impact on mitochondrial function and the expression of CREB/PGC-1α pathway proteins in mice with SCZ.

## Methods

### Experimental animals

In total, 30 adult male BALB/c mice (weighing 25–28 g) were obtained from Tabriz University of Medical Sciences (Tabriz, Iran) and kept under standard environments with a constant temperature (21.0 ± 2 °C), a 12-hour light/dark regimen, and free access to food and tap water. The research was carried out following the strict guidelines provided in the Guide for the Care and Use of Laboratory Animals by the National Institutes of Health (NIH; Publication No. 85 − 23, Revised 1985).

### Groups and treatment

**Fig. 1:**
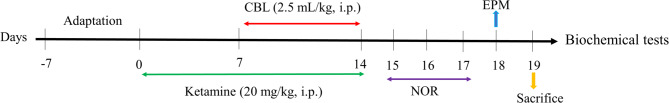
Timeline of the experiment study. NOR, novel object recognition; EPM, Elevated plus maze, and CBL, cerebrolysin

The mice were randomly assigned to three groups:

1. *Control group*: The mice included in this group received normal saline (1 mL/kg, 0.9% NaCl) via intraperitoneal (i.p.) injection for fourteen days.
2. *Ketamine group*: To induce the SCZ model, the animals received i.p. injections of ketamine at a dose of 20 mg/kg for fourteen consecutive days [33].
3. Ketamine + CBL group: In addition to receiving ketamine for fourteen days, between the 8th and 14th days of the study, the animals received daily i.p. injections of CBL (2.5 mL/kg, Ever Neuro Pharma, Unterach, Austria) [31]. (Fig. 1).

### Behavior tests

#### Novel object recognition test

The Novel Object Recognition (NOR) test, which leverages mice’s natural inclination to investigate new surroundings, is used to evaluate episodic-like memory. The task procedure consisted of three stages: habituation, training, and retention. In the habituation stage, the open-field arena, devoid of objects, was made available for exploration by each mouse for 10 min.

During the training stage, which was performed 24 h after habituation, each mouse was positioned in the same arena containing two identical objects for 10 min. After one day, the animal was situated in the previous arena, where two objects were present: one familiar and the other novel (retention stage). The discriminant index (DI) quantifies behavior by comparing exploration times of novel and familiar objects. It was calculated using the following equation to determine NOR. DI = (Time spent novel object − Time spent familiar object)/(Novel exploration time + Familiar exploration time) [34].

#### Elevated plus maze test

The mice underwent assessment for anxiety-like behavior using the elevated plus maze (EPM). The EPM setup consisted of four arms, with two open and two enclosed arms (50 cm in length × 10 cm in width), arranged perpendicularly and connected via a 5 × 5 cm central platform. The closed arms are enclosed by a transverse wall 40 cm in height. Each mouse was individually positioned on the central platform of the EPM apparatus and let to explore the arena freely for 6 min. The time spent in the open arms (OAT) and the number of open arms entries (OAE) were measured [35]. Following each session, a 70% ethanol solution was used to clean the mazes, thereby preventing odor cues. The tests were video-recorded from above using a camera installed over the mazes to monitor animal behavior, and analyzed using the ANY-Maze 7.4 tracking software.

### Sampling

After euthanasia of mice by cervical dislocation, the brains were quickly extracted from the skulls. The hippocampus was separated on an ice plate and then frozen in liquid nitrogen, after which it was stored at − 80 °C.

### Assessment of ROS generation

Hippocampal samples were homogenized in cold 40 mM Tris-HCl buffer, pH 7.4, and then incubated for half an hour at 37 °C with 25 µM of the fluorescent vital dye dichlorohydrofluorescein diacetate (DCFDA). Mitochondrial ROS oxidizes the fluorescent probe DCFDA, converting it into dichlorodihydrofluorescein (DCF). The fluorescence intensity was measured using a fluorescence microplate reader (λ excitation = 485 nm and λ emission = 530 nm). The ROS level was expressed as fluorescence intensity [34].

### Assessment of ATP levels

The ATP levels were measured in the hippocampal tissues with an ATP assay kit (MAK190, Sigma, USA), under the manufacturer’s protocol. Brain hippocampal tissues (10 mg) were homogenized in 100 mL of lysis ATP assay buffer. The ATP probe was added to the lysed homogenate along with the developer, and absorbance was read at 570 nm. Using an ATP standard curve, the ATP levels were determined, and the results were reported in picomoles per milligram of protein [36].

### Western blot

The levels of CREB, p-CREB, and PGC-1α proteins were assessed using Western blot analysis. Brain hippocampal tissues were homogenized in Radio Immuno Precipitation Assay lysis buffer (100 mL) with a protease inhibitor cocktail (Roche, Germany) to prevent protein degradation. Then, they were centrifuged at 12,000 g for 15 min at 4 °C. After electrophoresis on SDS-PAGE gels, the samples were blotted onto a PVDF membrane (Roche, UK). Subsequently, the membranes were blocked to inhibit non-specific binding, followed by overnight incubation with primary antibodies (Santa Cruz Biotechnology, USA) against CREB (sc-377154), p-CREB (sc-81486), and PGC-1α (sc-5815). Following three washes with PBS, the membranes were incubated for 2 h with horseradish peroxidase-conjugated anti-mouse IgG secondary antibody (sc-516102, 1:500). Using enhanced chemiluminescence Western blot detection reagents, protein bands were visualized and analyzed with ImageJ software (NIH, USA). GAPDH was employed as the internal control [32].

### Statistical analysis

Analysis of the data was accomplished using GraphPad Prism 9 software. Data were reported as mean ± SEM and analyzed by one-way ANOVA, followed by Tukey’s multiple comparison test. Additionally, the exploration time for each object in each group during the NOR test was analyzed using an unpaired two-tailed Student’s t-test. A p-value of less than 0.05 was considered statistically significant.

## Results

### NOR test

**Fig. 2:**
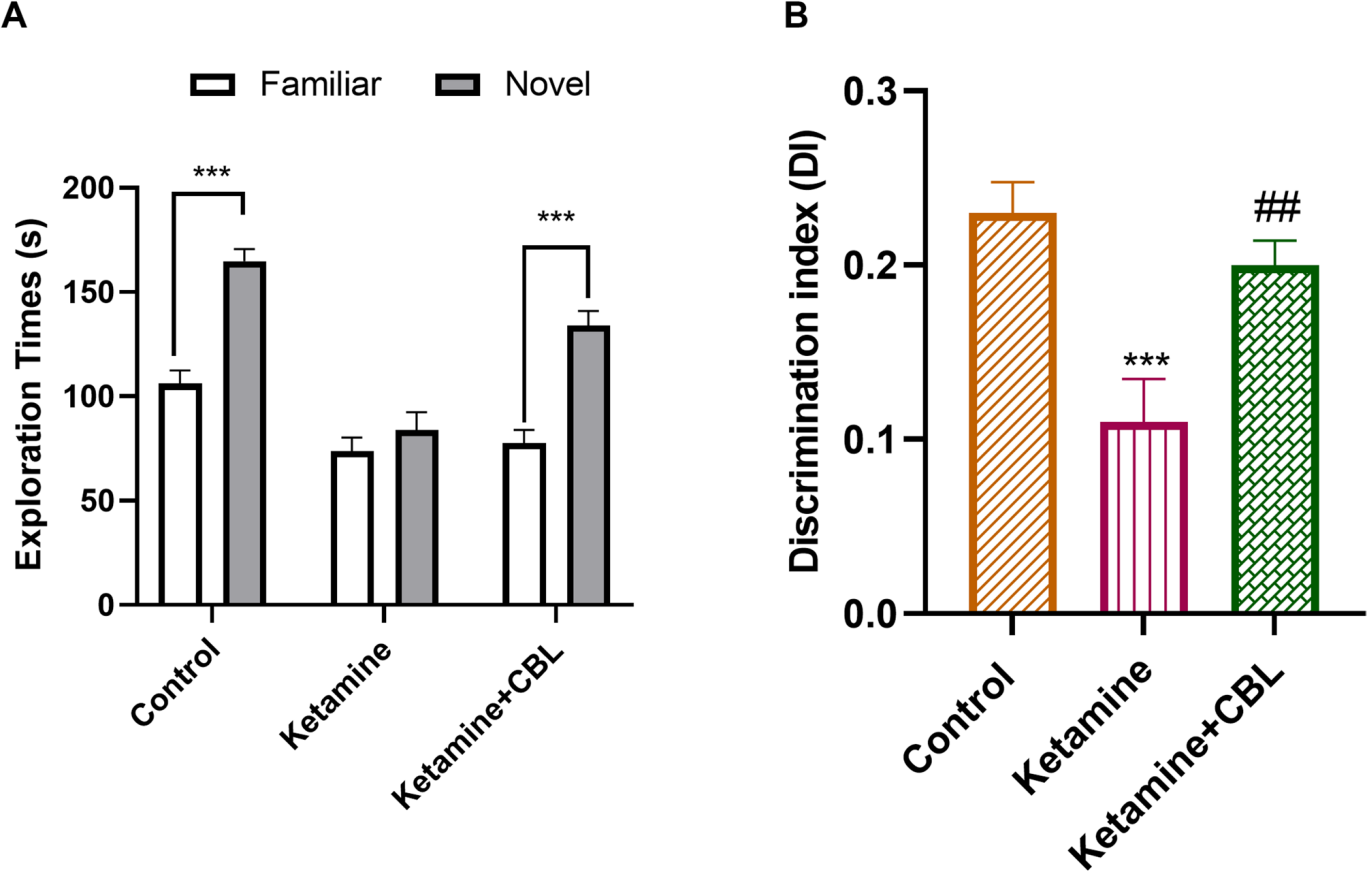
The effect of CBL on recognition memory in mice. **A** Exploration time of a familiar or novel object during the retention phase of the NOR test. Unpaired Student’s t-test, ***p < 0.001. **B** Discrimination index during the retention phase of the NOR test. Values are shown as mean ± SEM (*n* = 8 in each group). ***p < 0.001 compared to control group; ##*p* < 0.01 compared to Ketamine group. CBL, cerebrolysin; NOR, novel object recognition

The training session revealed no significant differences in object exploration time across groups (data not shown; *p* > 0.05). In the retention phase, all groups, except the ketamine group, spent more time exploring the new object than the familiar object (Fig. 2A, *p* < 0.001). In addition, the results of one-way ANOVA of DI displayed a significant difference between the study groups (F_(2, 21)_ = 10.40, *p* = 0.0007). Ketamine administration resulted in a significant decline in DI compared to control mice (*p* < 0.001). Notably, CBL treatment resulted in a considerable increase in DI in the mice in comparison with the ketamine group (*p* < 0.01, Fig. 2B).

### EPM test

**Fig. 3:**
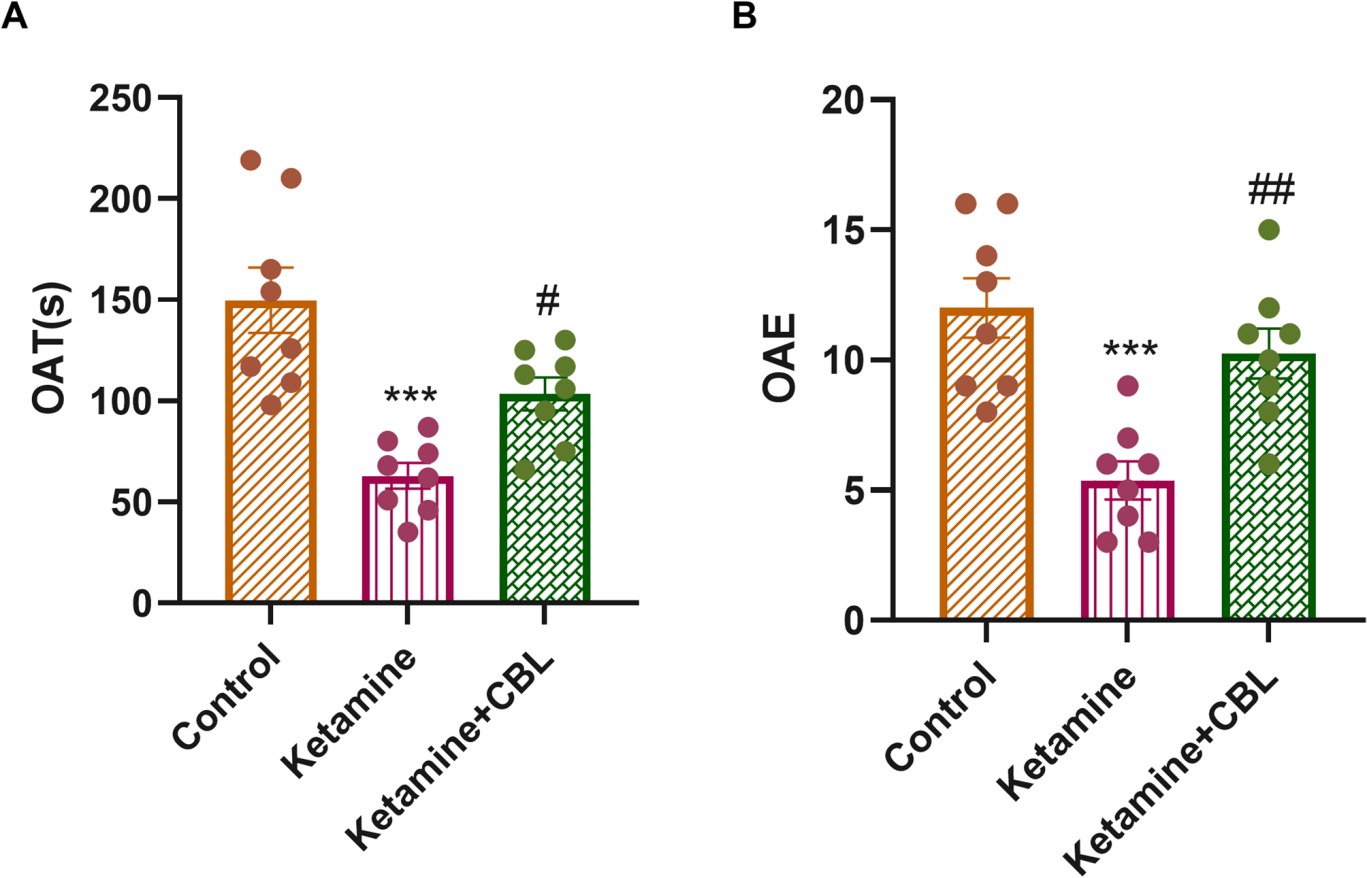
Effect of CBL on (**A**) the OAT and (**B**) OAE in the study groups. Values are presented as the means ± SEM (*n* = 8 in each group). ***p < 0.001 compared with control group; #*p* < 0.05 and ##*p* < 0.01 compared to Ketamine group. CBL, Cerebrolysin; OAE, open arms entries; OAT, open arms time

One-way ANOVA revealed statistically significant differences among the experimental groups in terms of OAT (F_(2, 21)_ = 15.40, *p* < 0.0001) and OAE (F_(2, 21)_ = 12.91, *p* = 0.0002). Figure 3A shows that the ketamine group spent a shorter period in the open arms compared to the control group (*p* < 0.01). Nevertheless, administration of CBL increased OAT in comparison with the ketamine group (*p* < 0.05). Moreover, the ketamine-treated mice had a significantly lower number of OAEs versus the mice in the control group (*p* < 0.001). A notable rise in OAE was detected in the Ketamine + CBL group compared to the ketamine group (*p* < 0.01, Fig. 3B).

### ROS and ATP levels

**Fig. 4:**
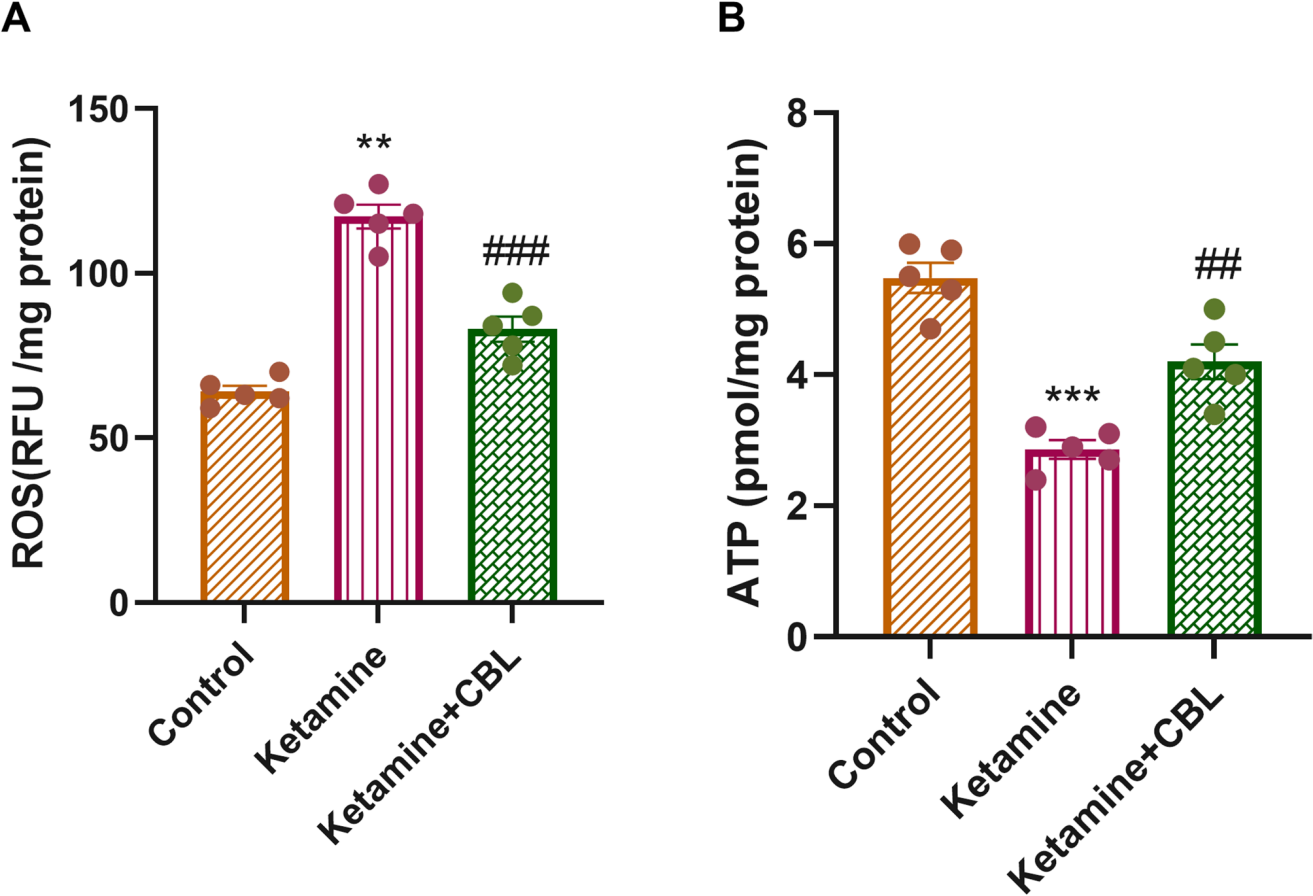
Effect of CBL on (**A**) ROS and (**B**) ATP levels in the hippocampus of ketamine-treated mice. Values are presented as mean ± SEM (*n* = 5 in each group). **p < 0.01, ***p < 0.001 compared to control group; ##*p* < 0.01 and ###*p* < 0.001 compared to Ketamine group. CBL, Cerebrolysin

A significant difference was observed in ROS levels (F_(2, 12)_ = 70.47, *p* < 0.0001) and ATP levels (F_(2, 12)_ = 35.27, *p* < 0.0001) among the study groups using one-way ANOVA. ROS levels were significantly elevated in ketamine-treated mice versus the control group (*p* < 0.01). CBL administration at 2.5 mL/kg to ketamine-treated mice significantly diminished the ROS levels versus the ketamine group (*p* < 0.001, Fig. 4A).

A significant reduction in ATP levels was detected in the hippocampus of ketamine-treated mice compared to control mice (*p* < 0.001), whereas treatment with CBL enhanced ATP levels relative to the ketamine-treated group (*p* < 0.01, Fig. 4B).

### Expression of PGC-1α, CREB, and P-CREB proteins

**Fig. 5:**
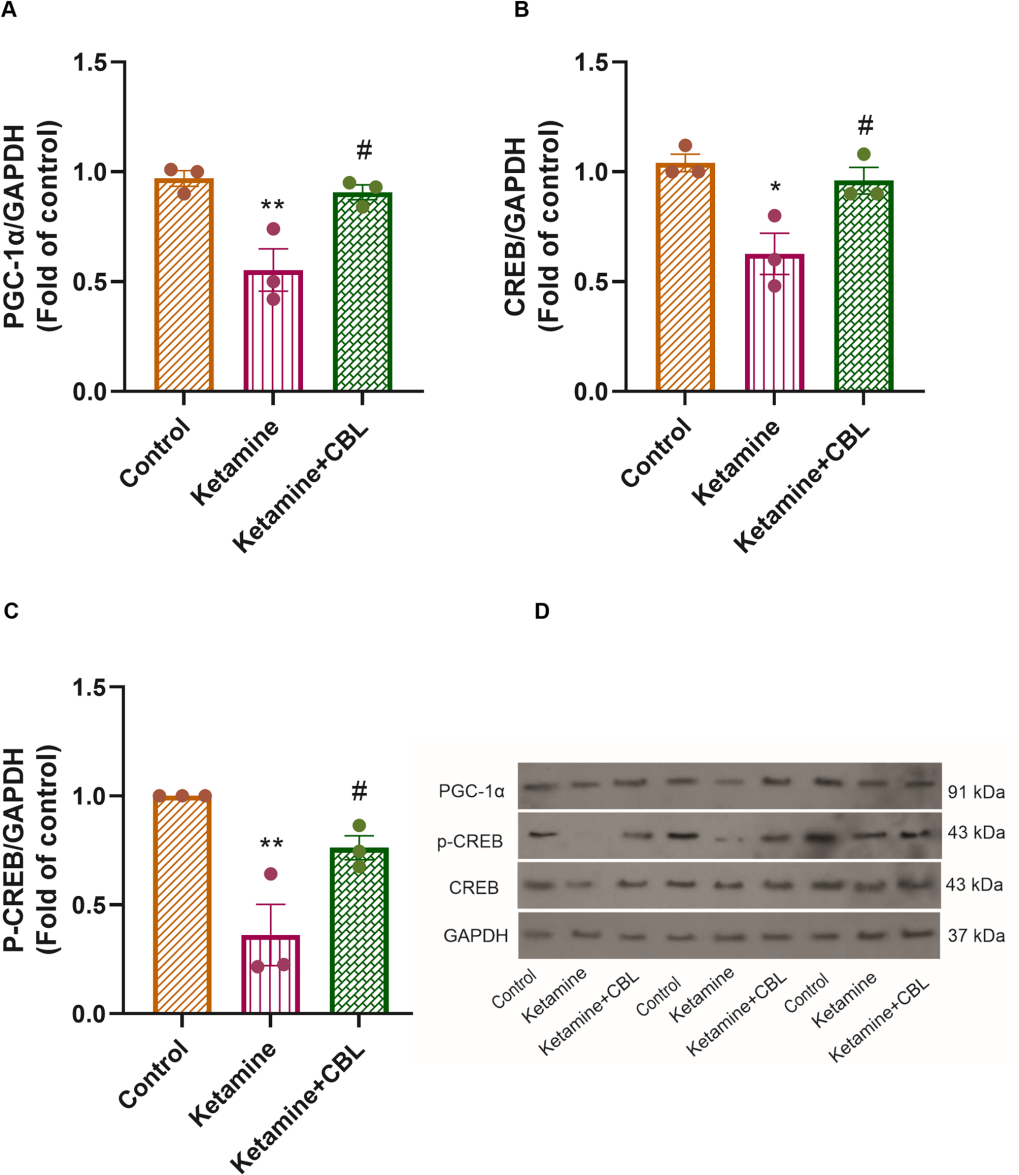
The effect of CBL on the expression of (**A**) PGC-1α, (**B**) CREB, and (**C**) p-CREB proteins in the hippocampus of the ketamine mice. (**D**) Representative blot images of proteins were established by immunoblotting. Values are presented as mean ± SEM (*n* = 3 in each group). *p < 0.05 and **p < 0.01 compared to the control group. #*p* < 0.05 compared to the ketamine group. CBL, Cerebrolysin

We also observed significant differences in hippocampal protein expression of PGC-1α (F_(2, 6)_ = 13.01, *p* = 0.0066), CREB (F_(2, 6)_ = 10.36, *p* = 0.0113), and p-CREB (F_(2, 6)_ = 13.74, *p* = 0.0058) among the study groups. As shown in Fig. 5A, the PGC-1α protein expression was reduced in the hippocampus region of ketamine-treated mice versus the control group (*p* < 0.01). CBL treatment upregulated PGC-1α expression versus the ketamine group (*p* < 0.05). Furthermore, the levels of CREB protein in the hippocampus of the ketamine group were diminished compared to those in the control group (*p* < 0.05). Treatment with CBL could restore the decline in CREB protein levels caused by ketamine (*p* < 0.05, Fig. 5B). Moreover, we found that the CBL significantly upregulated p-CREB expression in the hippocampus compared to the ketamine group (*p* < 0.05, Fig. 5C).

## Discussion

The findings of the current study, based on the NOR and EPM tests, showed that CBL administration in SCZ model mice improved cognitive functions and reduced anxiety-like behaviors. Additionally, CBL enhances mitochondrial function by decreasing ROS and increasing ATP levels, as well as by upregulating levels of PGC-1α, p-CREB, and CREB proteins in the hippocampus of these animals.

SCZ is a severe psychiatric condition characterized by a range of cognitive impairments, including deficits in memory, attention, and executive functioning, present in more than 80% of patients and meaningfully contributing to functional disability [37]. Cognitive impairments can be evidenced prior to the inception of psychosis, sometimes emerging in childhood or adolescence. Unlike positive symptoms, cognitive deficits in SCZ are largely unresponsive to existing antipsychotic treatments that primarily target dopamine D2 receptors [38]. Research on memory in individuals with SCZ has identified significant impairments in both verbal and visual aspects of recognition [39] and episodic memory [40]. In accordance with prior studies [33, 41], administration of ketamine at sub-anaesthetic doses caused memory impairments. In this study, mice treated with ketamine exhibited a decreased preference for exploring the new object in the NOR test, indicating memory deficits. Furthermore, ketamine administration in rodent models has been shown to increase anxiety-like behaviors, as demonstrated by increased locomotor activity in the open field test [42, 43]. Similarly, our findings showed that ketamine caused anxiety-like behavior in mice, as indicated by reduced time in the open arms during the EPM test.

CBL is a neuroprotective drug that mimics the effects of neurotrophic factors, such as BDNF and GDNF. It helps improve cognitive processes by increasing synaptic density and the integrity of neuronal circuits [30]. It has potential for the treatment of neuropsychiatric diseases, including SCZ [44]. The results of a clinical study showed that administering CBL as an adjunctive medication to patients with SCZ reduced negative symptoms and improved memory and cognitive functions, with no side effects reported from CBL use in these patients [45]. Additionally, an animal study found that CBL can reduce responsiveness to novel environments, decrease amphetamine-induced movements, and enhance social interactions in the SCZ model by mitigating damage to dendritic neurons in the limbic region [30]. CBL has improved cognitive function in patients with vascular dementia and Alzheimer’s disease [46]. Likewise, CBL treatment improved memory performance in the NOR test and reduced anxiety signs in mice treated with ketamine.

The findings of this investigation indicate that CBL improves recognition memory in the NOR task, which can be characterized by its actions across the three stages of memory: Encoding, consolidation, and retrieval. CBL enhances synaptic plasticity and promotes the generation of new neurons, both of which are essential for the proper encoding [47]. The protein CREB functions as a molecular switch for memory formation, and its upregulation has been shown to enhance memory consolidation [48]. In the current study, CBL increased hippocampal p-CREB, possibly related to memory consolidation in the NOR paradigm. In addition, CBL increases neurotrophic factors and mitochondrial function, which can help stabilize and strengthen synaptic connections [47, 49, 50]. The retrieval phase refers to the process of accessing stored information, which in the NOR paradigm is manifested in the animal’s ability to discriminate between the new and the familiar object, thereby demonstrating the functional integrity of recognition memory [51]. We have also shown that CBL treatment has a significant effect on retrieval memory.

There is considerable evidence that impaired mitochondrial function is involved in the development and progression of SCZ [52–54]. Alterations in mitochondrial dynamics, triggered by redox changes, contribute to the development of chronic inflammation and oxidative/nitrosative stress, leading to immune-inflammatory responses and progressive neurological changes [16]. Mitochondrial hypoplasia in SCZ disrupts neuronal plasticity through inflammation, oxidative stress, and changes in ATP and cytoplasmic calcium levels, resulting in changes in hippocampal circuitry, episodic memory deficits, and impaired peripheral interactions [52]. Furthermore, it has been demonstrated that genetic changes in mitochondria occur in SCZ, which are directly linked to a reduction in dendritic spines and alterations in dendritic architecture in the neocortex [55]. Our findings revealed a significant increase in ROS concentrations and a decrease in ATP levels in the hippocampus of mice treated with ketamine. Conversely, CBL treatment was able to restore both ROS and ATP levels. Consistent with our findings, the antioxidant effects of CBL have been demonstrated in multiple studies [56, 57].

It has been revealed that PGC-1α is a key factor in the formation and maintenance of dendritic spines in the striatum, pyramidal neurons, and hippocampus [58]. Decreased expression of PGC-1α-related genes disrupts neuronal circuits in these areas, leading to cognitive and motor disorders, as well as neurological diseases [59]. A recent study has demonstrated that enhanced mitochondrial biogenesis through the CREB/PGC-1α pathway results in activation of PGC-1α, NRF-1, TFAM, and phosphorylation of CREB [60]. Improving mitochondrial function increases CREB levels by increasing intracellular cAMP levels [61]. It has been demonstrated that increasing the expression of factors involved in mitochondrial biogenesis, such as PGC-1α, NRF1, and TFAM, enhances antioxidant capacity and reduces neurodegeneration, neuronal death, and neurobehavioral deficits in an animal model [62]. A study on an animal model of Parkinson’s has shown that activation of the CREB/PGC-1α pathway has a neuroprotective effect on dopaminergic neurons, protecting them from apoptosis [63]. We also found a reduction in PGC-1α expression in the hippocampus of mice treated with ketamine, while treatment with CBL could upregulate its expression.

Importantly, studies indicate that CREB is involved in signaling pathways related to the development and treatment of mental disorders, including SCZ, making CREB a key focus of research. Abnormal CREB expression has been observed in the brains of individuals with SCZ [64]. It has been reported that CREB dysfunction in the hippocampus, through the downregulation of interleukin-2, causes cognitive deficits and depressive-like behaviors in mice [65]. Luo and colleagues found that chronic ketamine administration suppressed the protein expression and phosphorylation of CaMKIIβ, ERK1/2, CREB, and NF-κB, leading to cognitive impairments [66]. In agreement with previous research, our results showed that ketamine decreased CREB levels in the hippocampus and was associated with cognitive dysfunction in mice. Despite this, the study revealed that the CBL treatment upregulated CREB and p-CREB expression in the hippocampal tissue of mice treated with ketamine.

The findings of a research revealed that CBL, by affecting the CREB/PGC-1α pathway and increasing levels of CREB and PGC-1α proteins, suppresses inflammatory gene expression and enhances anti-inflammatory gene expression in rats [32]. CBL reduces TNF-α, iNOS, and caspase-3 in the brain [56]. By reducing oxidative stress and inflammation and improving mitochondrial function, it has potential as a therapeutic option for treating neurological diseases [56, 67, 68].

There are several limitations in the current study that should be noted. Our experiments were conducted solely with male mice. Future research that incorporates both male and female subjects will be crucial to ascertain whether the neuroprotective effects of CBL observed in the present study are also applicable to female subjects. Moreover, the duration of CBL administration was limited to 7 days. Further investigations are required to evaluate the prolonged efficacy and safety of CBL. Another limitation is the small sample size in the Western blot, due to financial constraints.

Summing up, the present study outcomes revealed that CBL could reduce cognitive deficits and anxiety-like behaviors in a mouse model of SCZ by activating the CREB/PGC-1α pathway and improving mitochondrial function. Further research is essential to identify other mechanisms that may contribute to CBL’s effects on SCZ-like symptoms in animals.

## Supplementary Information

Below is the link to the electronic supplementary material.

Supplementary Material 1

Supplementary Material 2

Supplementary Material 3

## Data Availability

The data will be made available upon request.
