# Supplementary figures and images for: Cerebrolysin ameliorates ketamine-mediated anxiety and cognitive impairments via modulation of mitochondrial function and CREB/PGC-1α pathway
Source: Mol Brain. 2025 Nov 7;18:84. doi: 10.1186/s13041-025-01255-1 (PMC12595893; doi:10.1186/s13041-025-01255-1)

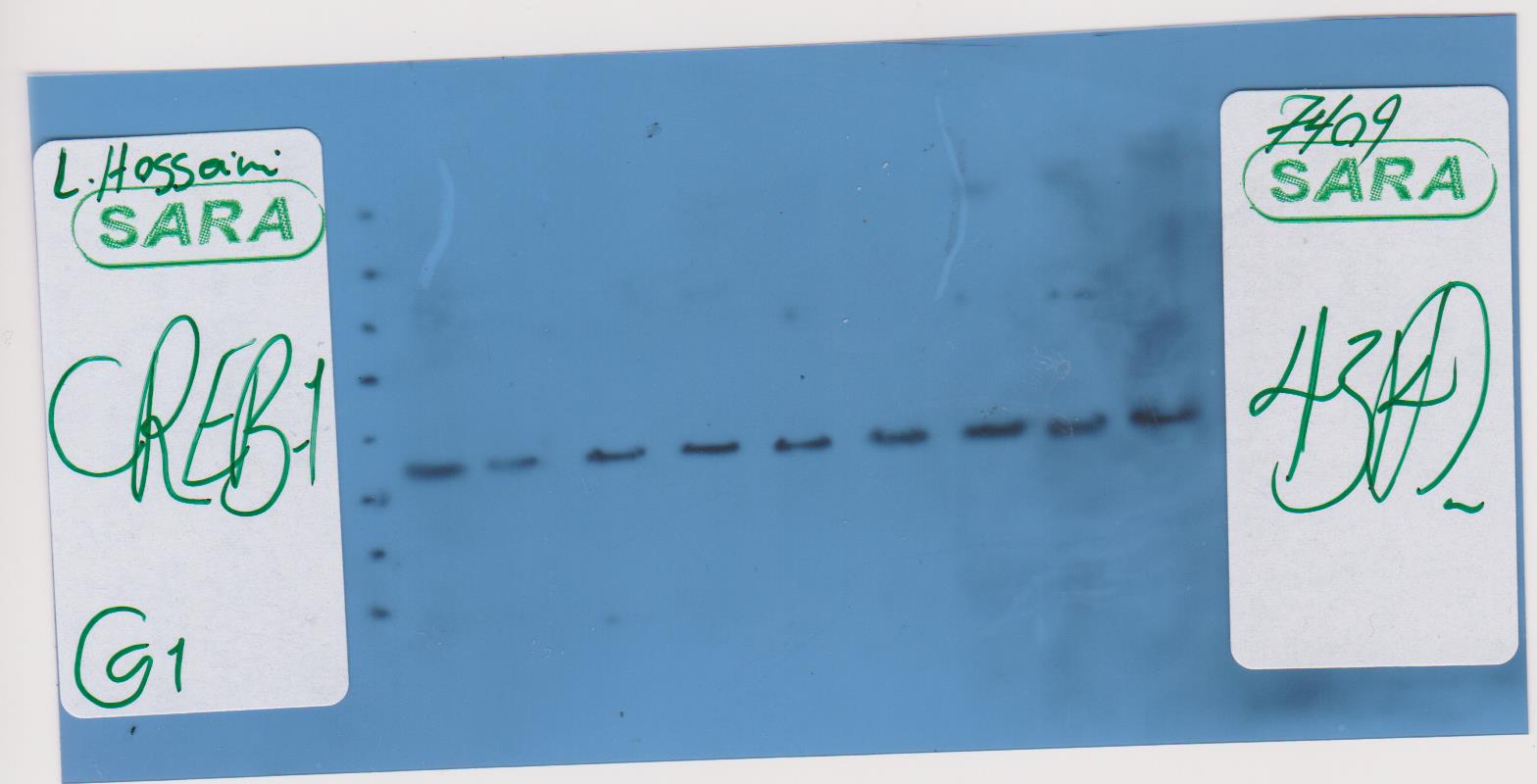

Supplement: Supplementary file 1 — Supplementary Material 1 [file 13041_2025_1255_MOESM1_ESM.zip › original images/colored images/CREB-1(G1).jpg]

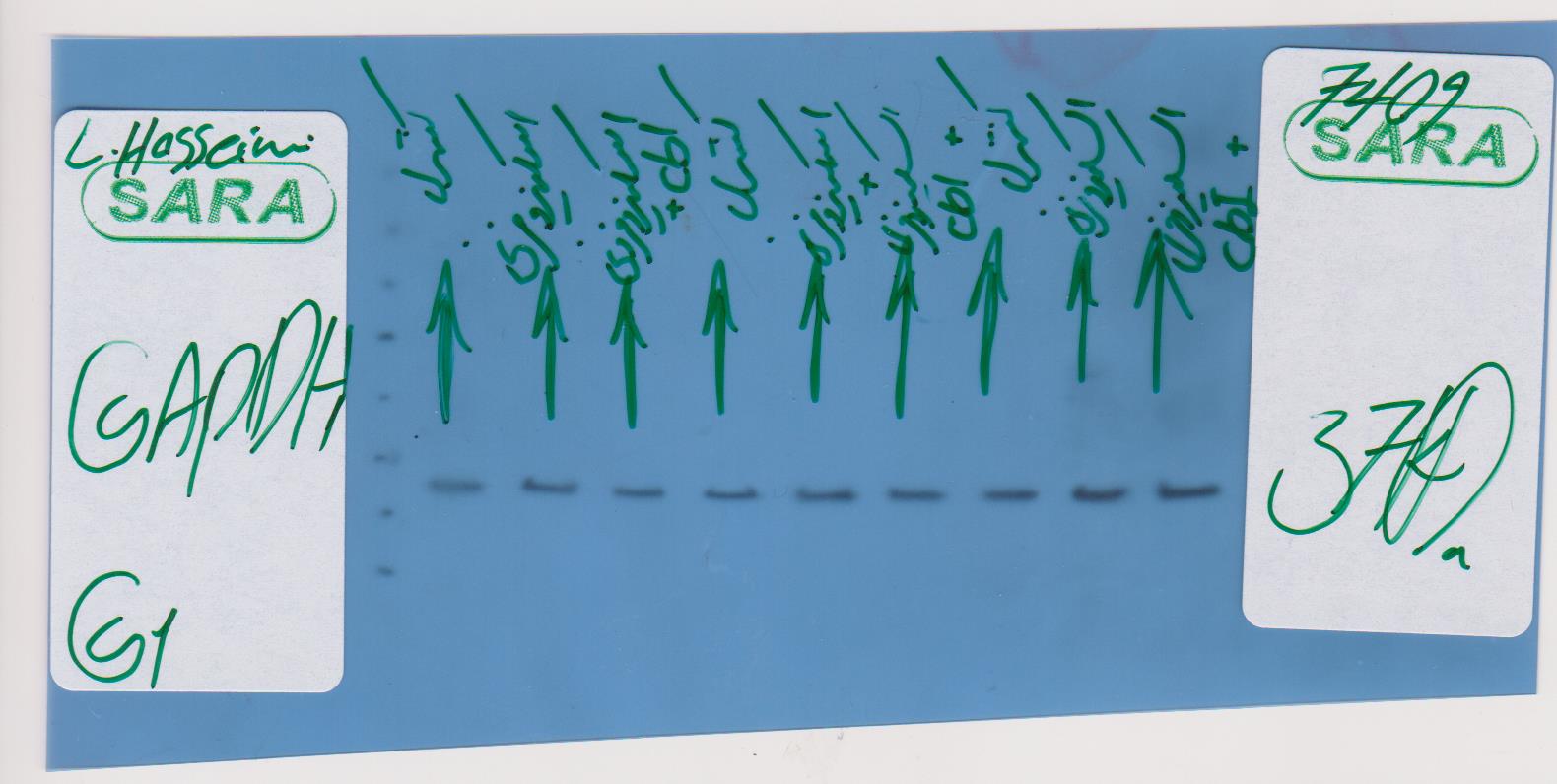

Supplement: Supplementary file 1 — Supplementary Material 1 [file 13041_2025_1255_MOESM1_ESM.zip › original images/colored images/GAPDH(G1).jpg]

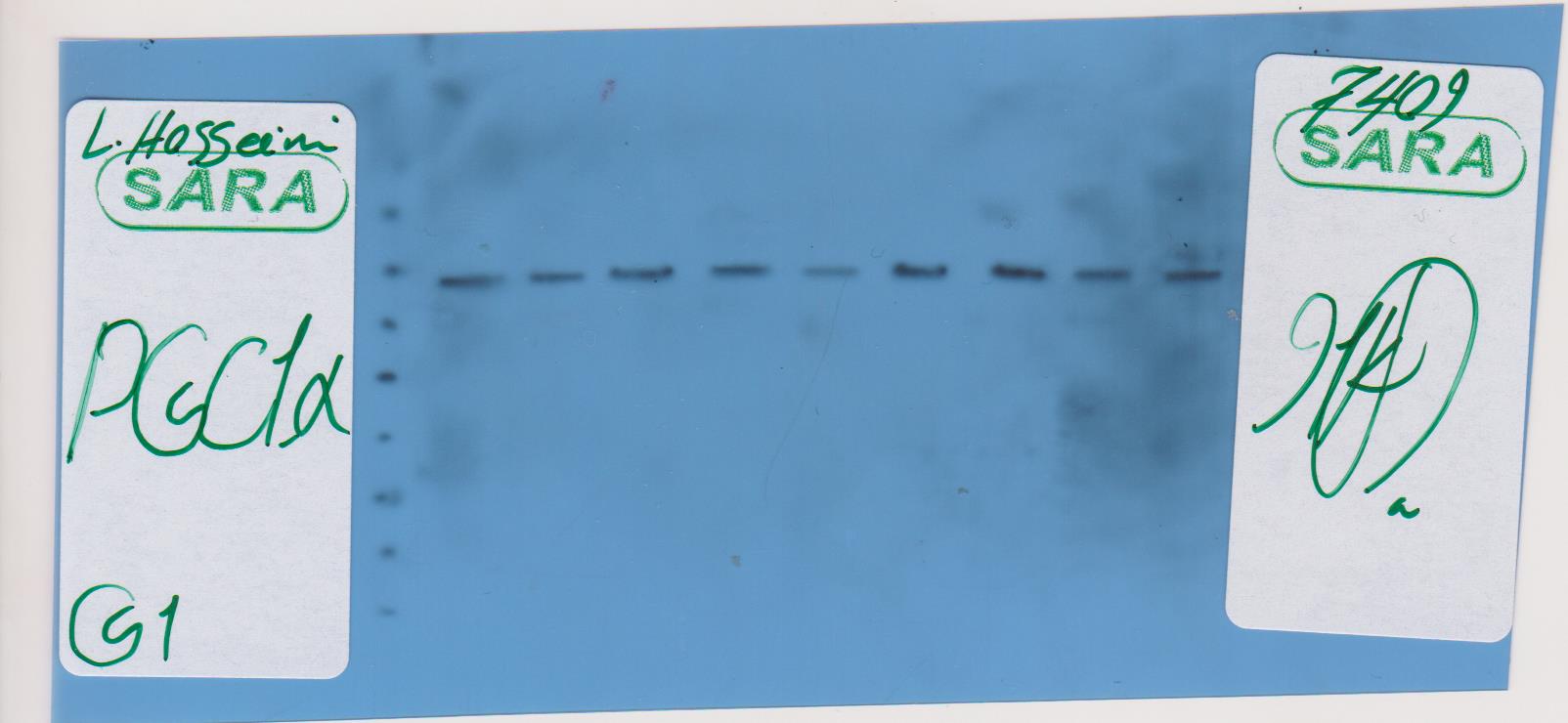

Supplement: Supplementary file 1 — Supplementary Material 1 [file 13041_2025_1255_MOESM1_ESM.zip › original images/colored images/PGC1a(G1).jpg]

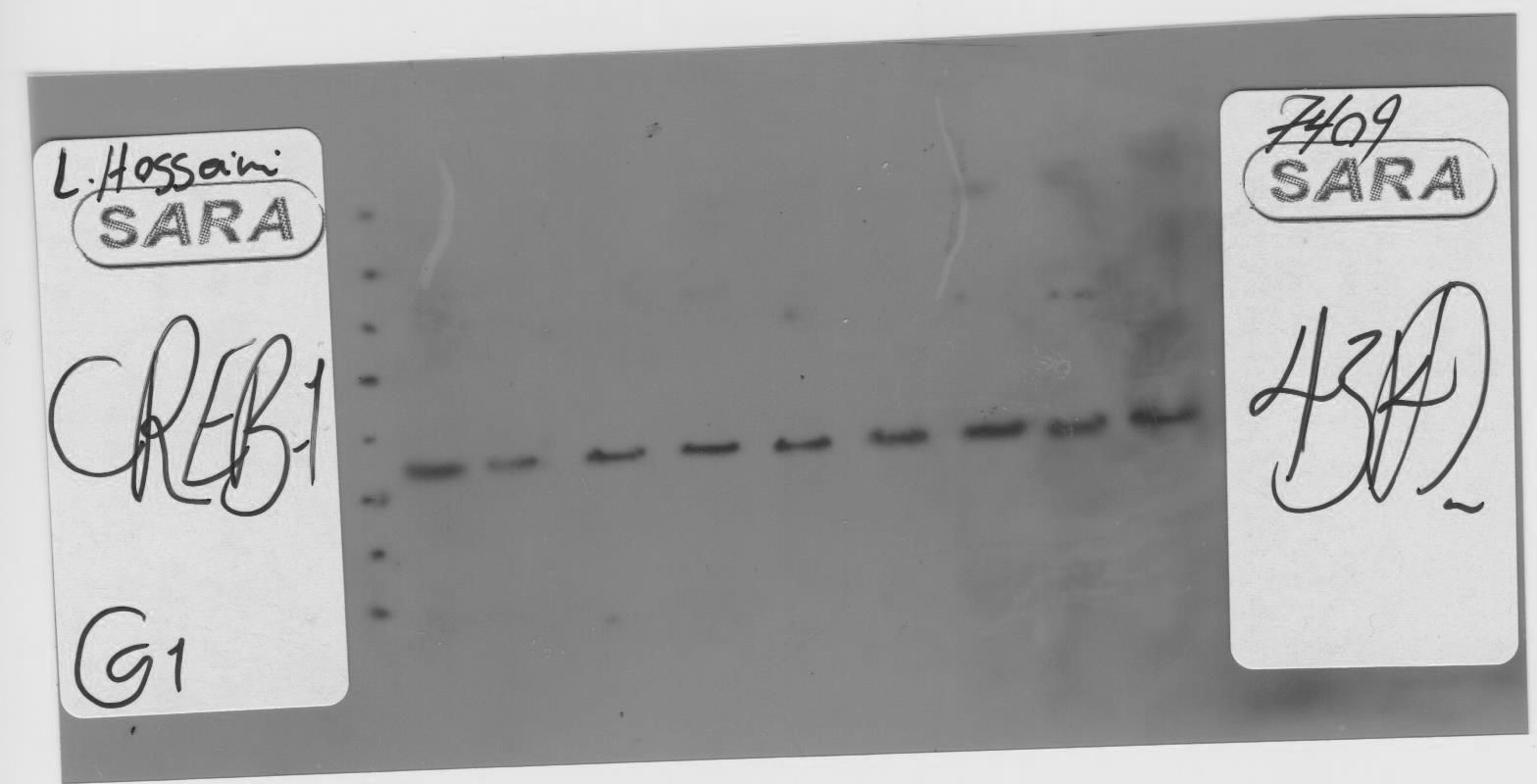

Supplement: Supplementary file 1 — Supplementary Material 1 [file 13041_2025_1255_MOESM1_ESM.zip › original images/gray scaled images/CREB-1(G1).jpg]

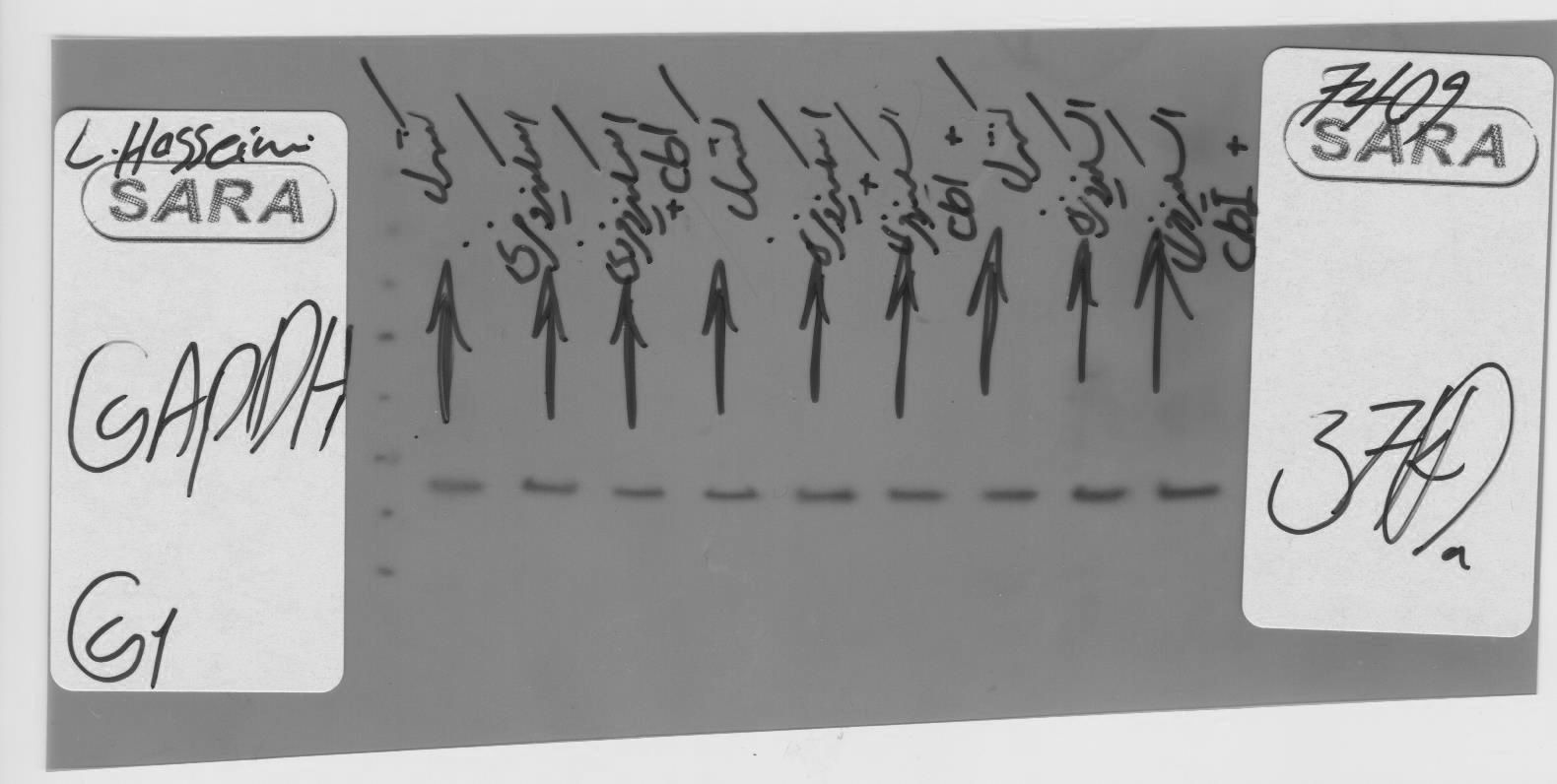

Supplement: Supplementary file 1 — Supplementary Material 1 [file 13041_2025_1255_MOESM1_ESM.zip › original images/gray scaled images/GAPDH(G1).jpg]

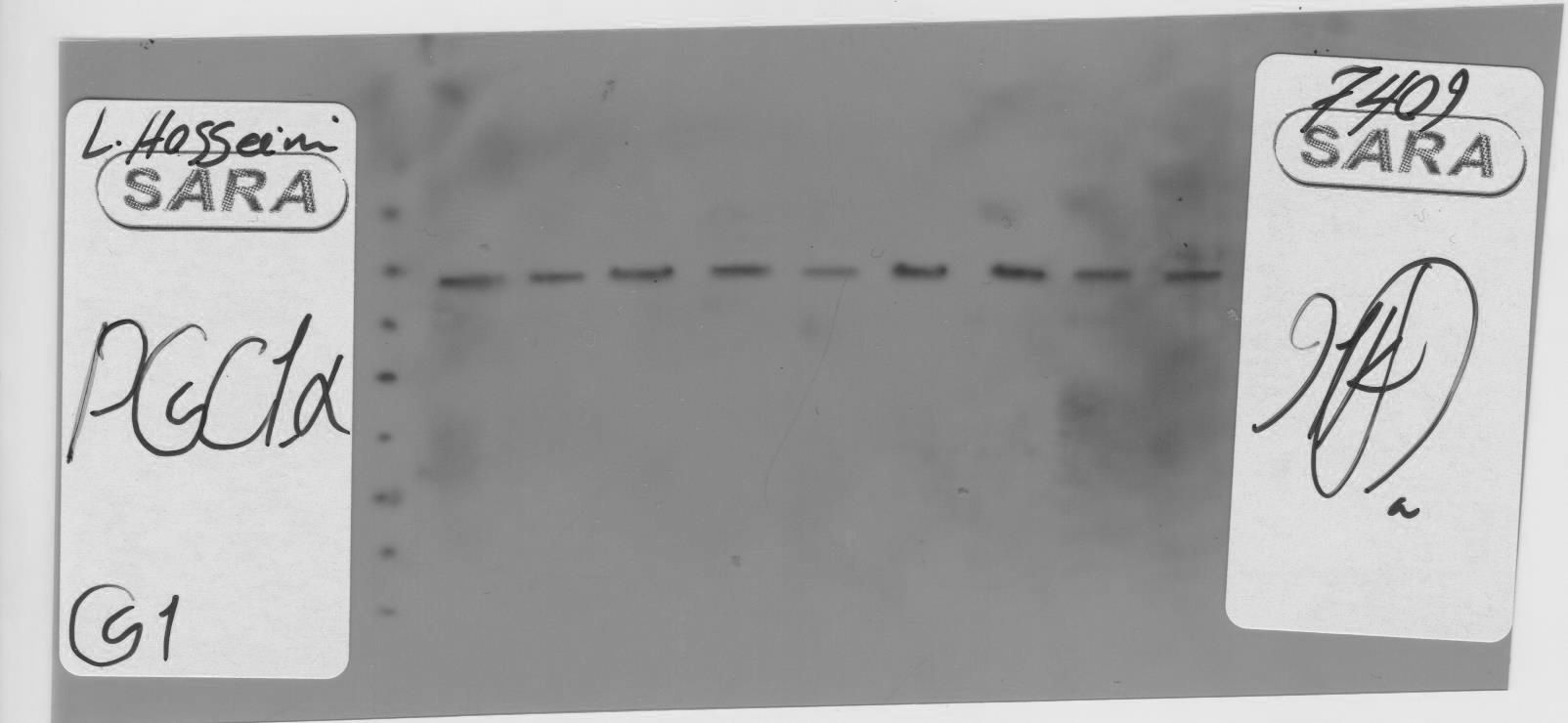

Supplement: Supplementary file 1 — Supplementary Material 1 [file 13041_2025_1255_MOESM1_ESM.zip › original images/gray scaled images/PGC1a(G1).jpg]

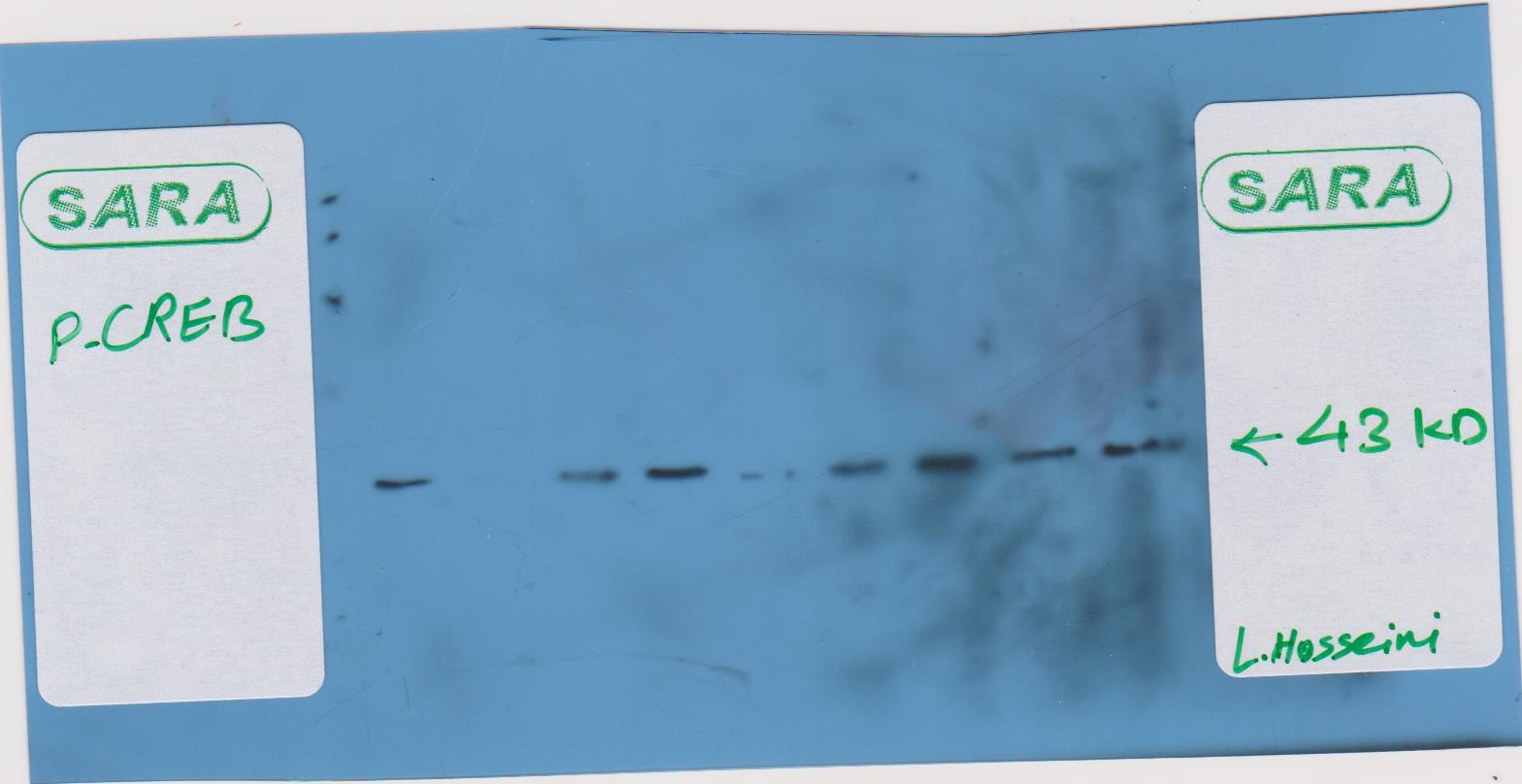

Supplement: Supplementary file 3 — Supplementary Material 3 [file 13041_2025_1255_MOESM3_ESM.zip › original images western blot/colored images/p-CREB.jpg]

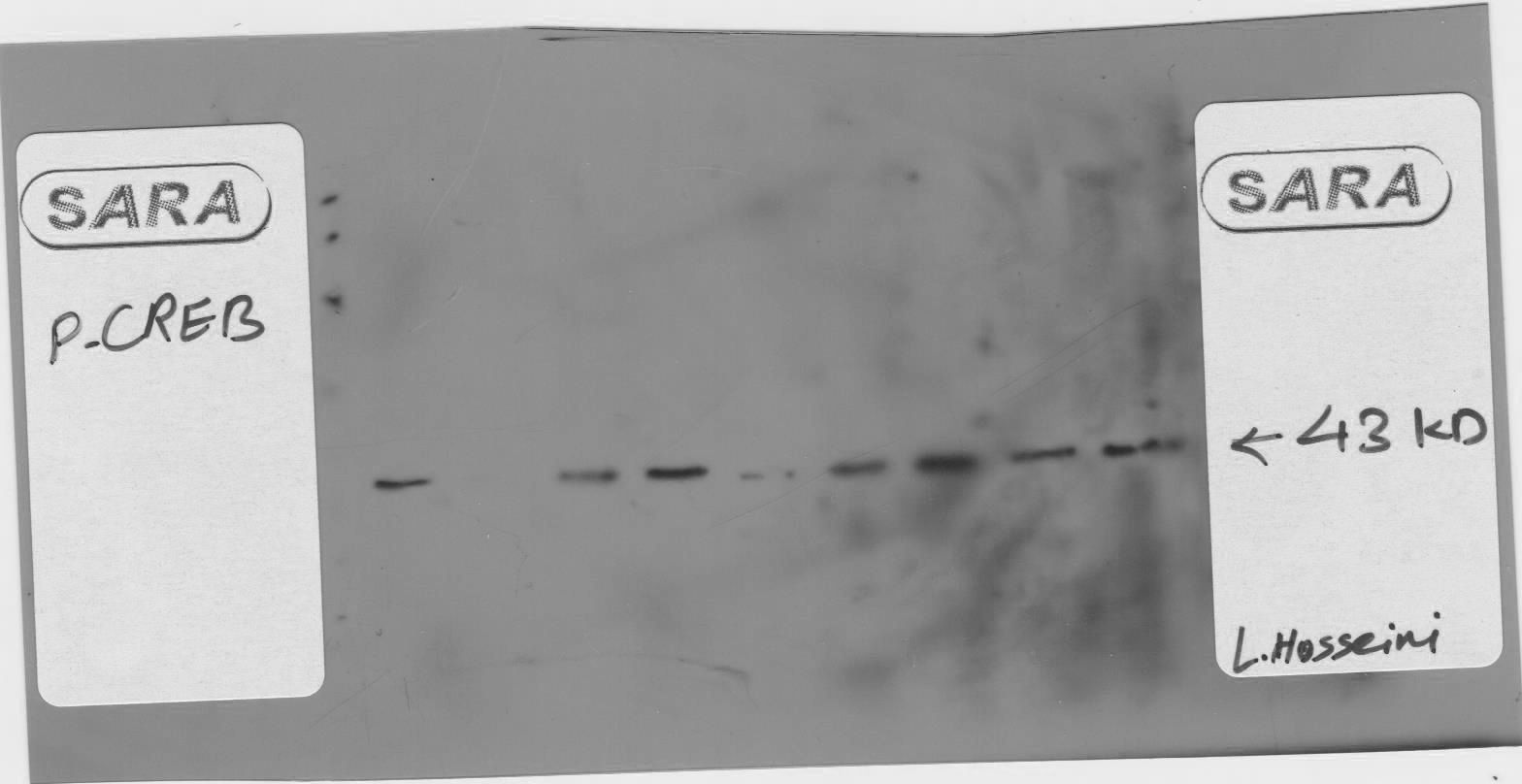

Supplement: Supplementary file 3 — Supplementary Material 3 [file 13041_2025_1255_MOESM3_ESM.zip › original images western blot/gray scaled images/p-CREB.jpg]
